# Diverse Mucosal-Associated Invariant TCR Usage in HIV Infection

**DOI:** 10.4049/immunohorizons.2100026

**Published:** 2021-05-27

**Authors:** Shubhanshi Trivedi, Taliman Afroz, Michael S. Bennett, Kendal Angell, Fabio Barros, Racheal A. Nell, Jian Ying, Adam M. Spivak, Daniel T. Leung

**Affiliations:** * Division of Infectious Disease, Department of Internal Medicine, University of Utah, Salt Lake City, UT;; † Division of Microbiology and Immunology, Department of Pathology, University of Utah, Salt Lake City, UT

## Abstract

Mucosal-associated invariant T (MAIT) cells are innate-like T cells that specifically target bacterial metabolites but are also identified as innate-like sensors of viral infection. Individuals with chronic HIV-1 infection have lower numbers of circulating MAIT cells compared with healthy individuals, yet the features of the MAIT TCR repertoire are not well known. We isolated and stimulated human PBMCs from healthy non-HIV–infected donors (HD), HIV-infected progressors on antiretroviral therapy, and HIV-infected elite controllers (EC). We sorted MAIT cells using flow cytometry and used a high-throughput sequencing method with bar coding to link the expression of TCRα, TCRβ, and functional genes of interest at the single-cell level. We show differential patterns of MAIT TCR usage among the groups. We observed expansions of certain dominant MAIT clones in HIV-infected individuals upon *Escherichia coli* stimulation, which was not observed in clones of HD. We also found different patterns of CDR3 amino acid distributions among the three groups. Furthermore, we found blunted expression of phenotypic genes in HIV individuals; most notably, HD mounted a robust *IFNG* response to stimulation, whereas both HIV-infected progressors and EC did not. In conclusion, our study describes the diverse MAIT TCR repertoire of persons with chronic HIV-1 infection and suggest that MAIT clones of HIV-infected persons may be primed for expansion more than that of noninfected persons. Further studies are needed to examine the functional significance of unique MAIT cell TCR usage in EC.

## INTRODUCTION

Despite the great advances of antiretroviral therapy (ART) in the effective control of HIV replication, it is not curative. With 36.9 million people living with HIV globally (UNAIDS.org; http://www.unaids.org/en/resources/fact-sheet), it remains a global public health problem. Sexual transmission is the main route of transmission of HIV (1, 2). The female genital tract consisting of the upper reproductive tract (uterus and cervix) and the lower reproductive tract (vagina) constitutes the major port of entry of several pathogens, such as HIV (2, 3). GALT is also an important portal of HIV-1 viral entry, active replication, and CD4^+^ T cell depletion (4–6). Ninety percent of infections occur at mucosal surfaces (7). It is well established that an effective immune response at these mucosal surfaces is needed to control HIV-1 (8).

Mucosal-associated invariant T (MAIT) cells are nonclassical innate-like T cells that are highly abundant in mucosal tissues, liver, and circulation of healthy humans (9–11). They play an important role in controlling bacterial infections (12–16) and are activated during human viral infections (17). MAIT cells express a semi-invariant αβ TCR that recognizes riboflavin metabolites presented by the nonpolymorphic MHC–like molecule MR1 (18, 19). Human MAIT TCR is composed of an invariant TCR α-chain TRAV1–2 with TRAJ33 (or TRAJ12/TRAJ20 at lower frequencies) paired with varied TCR b-chain, predominantly TRBV20 and TRBV6 (20, 21). MAIT TCR β-chain repertoire is more diverse, with diversity residing within the CDR 3β loop, which may facilitate differential Ag recognition (22–24). After Ag recognition, in either a TCR-dependent or TCR-independent manner (25), the activation of MAITs leads to secretion of proinflammatory cytokines, including IFN-γ, TNFα, IL-17, and other factors, resulting in lysis of the infected cells (26).

Although MAIT cells do not directly recognize HIV Ags, it is believed that they are uniquely armed to aid in the clearance of HIV-1–infected cells at mucosal surfaces (27). In addition, the polymicrobial reactivity and breadth of the MAIT cell functional profile most likely contribute to the reported role of MAIT cells in the protection against bacterial infections, including pulmonary tuberculosis in humans (28–30). MAIT cell deficiencies in HIV-1 patients may therefore increase their risk to secondary microbial coinfections (31, 32). MAIT cells are severely reduced and are functionally exhausted in HIV-infected patients, and generally, levels of MAIT cells in peripheral blood do not recover in response to successful combination ART (33–35). Such reductions in circulating frequency could be a result of their migration into mucosal tissue or, alternatively, due to an inability for MAIT cell populations to clonally expand or proliferate. Little is known regarding the clonality of MAIT cells in chronic HIV infection, particularly among individuals who are able to endogenously control HIV replication and maintain normal T cell counts in the absence of ART (known as elite controllers [EC]). In addition, prior studies of MAIT TCR usage have analyzed separately TCRα and TCRβ usage (24, 27), and there is a paucity of data on paired usage, necessary to optimally examine clonality.

In this study, using single-cell bar-coded sequencing linking TCR and functional genes (36), we investigated whether the MAIT TCR clonal distribution and phenotype are unique in HIV-infected individuals, especially in those who are EC, compared with HIV-infected progressors (PR; who are aviremic) and healthy non-HIV–infected donors (HD). We show that MAIT cells have a unique clonal distribution in EC compared with PR and HD.

## MATERIALS AND METHODS

### Participants

Aviremic HIV-1–infected progressors on ART and EC were recruited for phlebotomy according to an approved and active institutional review board protocol at the University of Utah (IRB_0058246), as described previously (37). Informed consent and phlebotomy were performed in the Center for Clinical and Translational Science Clinical Services Core at the University of Utah Medical Center. Participant characteristics are provided in Table 1. Healthy donor PBMCs were obtained via peripheral phlebotomy according to a separate approved and active institutional review board protocol at the University of Utah (IRB_0067637).

### Single-cell sorting and flow cytometry

To stimulate MAIT cells, PBMCs were stimulated with *Escherichia coli* strain 1100–2 (provided by the Coli Genetics Stock Center [Yale]) overnight. We spun down thawed aliquots of *E. coli*, fixed them in 1% paraformaldehyde in PBS for 10 min at room temperature, and washed them twice in PBS immediately prior to addition to cells at multiplicity of infection of 100. Following stimulation, cells were surface stained with CD3 PerCP/Cy5.5 (Clone OKT3; BioLegend), Vα 7.2 PE (clone 3C10; BioLegend), CD161 allophycocyanin (clone HP-3G10; Tonobo), and CD19 FITC (clone HIB19; BioLegend) at 4°C for 25 min. CD19^−^CD3^+^ Va7.2^+^ CD161^+^ MAIT cells were single-cell sorted into an RT-PCR buffer in a 96-well plate using BD FACSAria cell sorter. We sorted a total of 3072 MAIT cells from all subjects (~100–150 MAIT cells per subject, per stimulation condition).

### MAIT TCR sequencing and phenotyping

We next used a high-throughput sequencing method with bar coding to link the expression of TCRα, TCRβ, and functional genes of interest at the single-cell level as described in Han et al. (36). Briefly, for first reaction, reverse transcription and preamplification were performed with New England Biolabs (NEB) OneTaq One-Step RT-PCR Kit using V α and V β region primers, C region primers, and phenotyping primers in 10-μl reaction. For PCR no. 1, the final concentration of each TCR V region primer is 0.06 μM, each C region primer is 0.3 μM, and each phenotyping primer is 0.1 μM. A 25-cycle first RT-PCR was done per manufacturer’s instructions using the following cycling conditions: 48°C for 40 min, 94°C for 1 min, 25 cycles of 94°C for 15 s, 62°C for 1 min, and 68°C for 1 min followed by 68°C for 5 min and 4°C hold. Next, a 1-μl aliquot of the first reaction was used as a template for second 15-μl PCR for either TCR sequencing or phenotyping using NEB One Taq Hot Start DNA polymerase kit. The cycling conditions were as follows: 94°C for 30 s, followed by 94°C for 15 s, 64°C for 1 min, 68°C for 1 min for 25 cycles (for TCR) or 35 cycles (for phenotyping), and final 68°C for 5 min and 4°C hold. For the third 15-μl PCR, which incorporates barcodes and enables Illumina MiSeq sequencing, 1 μl of second PCR product was used as a template, and amplification was performed using NEB One Taq Hot Start DNA polymerase, 5’ and 3’ barcoding primers (0.375 μM each), and Illumina Paired-End primers (0.5 μM). The cycling conditions were 94°C for 30 s, followed by 36 cycles of 94°C for 15 s, 66°C for 30 s, 68°C for 1 min, and final 68°C for 5 min and 4°C hold. The PCR products were combined at equal proportion by volume (5 μl) and run on a 1.2% agarose gel, and a band around 375 bp was excised and gel purified using a Qiaquick gel extraction kit (Qiagen). This purified product was then sequenced. For all primer sequences, kindly refer to Han et al. (36).

### Sequencing data analysis

To separate reads from every well in every plate according to specified barcodes, we processed and demultiplexed raw sequencing data using a custom software pipeline as described in Han et al. (36). For reporting a cytokine or chain, we establish a threshold normalized depth of 50%. Minimum percentage of dominance for asserting β-chain and first α-chain was also set to 50%. The data were analyzed using R package. The code and VDJ pipeline have been deposited online (https://github.com/LeungLab/TcellUsageHIV).

### Statistical analysis

For MAIT cell phenotypic analysis, significant differences between HD, PR, and EC were assessed using two-way ANOVA and Tukey multiple comparisons test. Statistical analyses were performed using Prism Version 8 software (Graph-Pad), and *p* values <0.05 were considered significant.

To describe diversity, entropy for distribution of amino acid at each of the 10 sites of each of three groups (HD, EC, PR) before and after stimulation was calculated as described (38). Sequence similarity between each pair of subjects of each pair of groups and within each group among the three groups (HD, EC, PR) before and after stimulation was calculated as described (W.-J. Shen, H.-S. Wong, Q.-W. Xiao, X. Guo, and S. Smale, manuscript posted on arXiv, 1205.6031). The mean similarity is then calculated for each appropriate scope.

## RESULTS

### Diversity of TCR usage in MAIT cells in HIV infection

**FIGURE 1.**
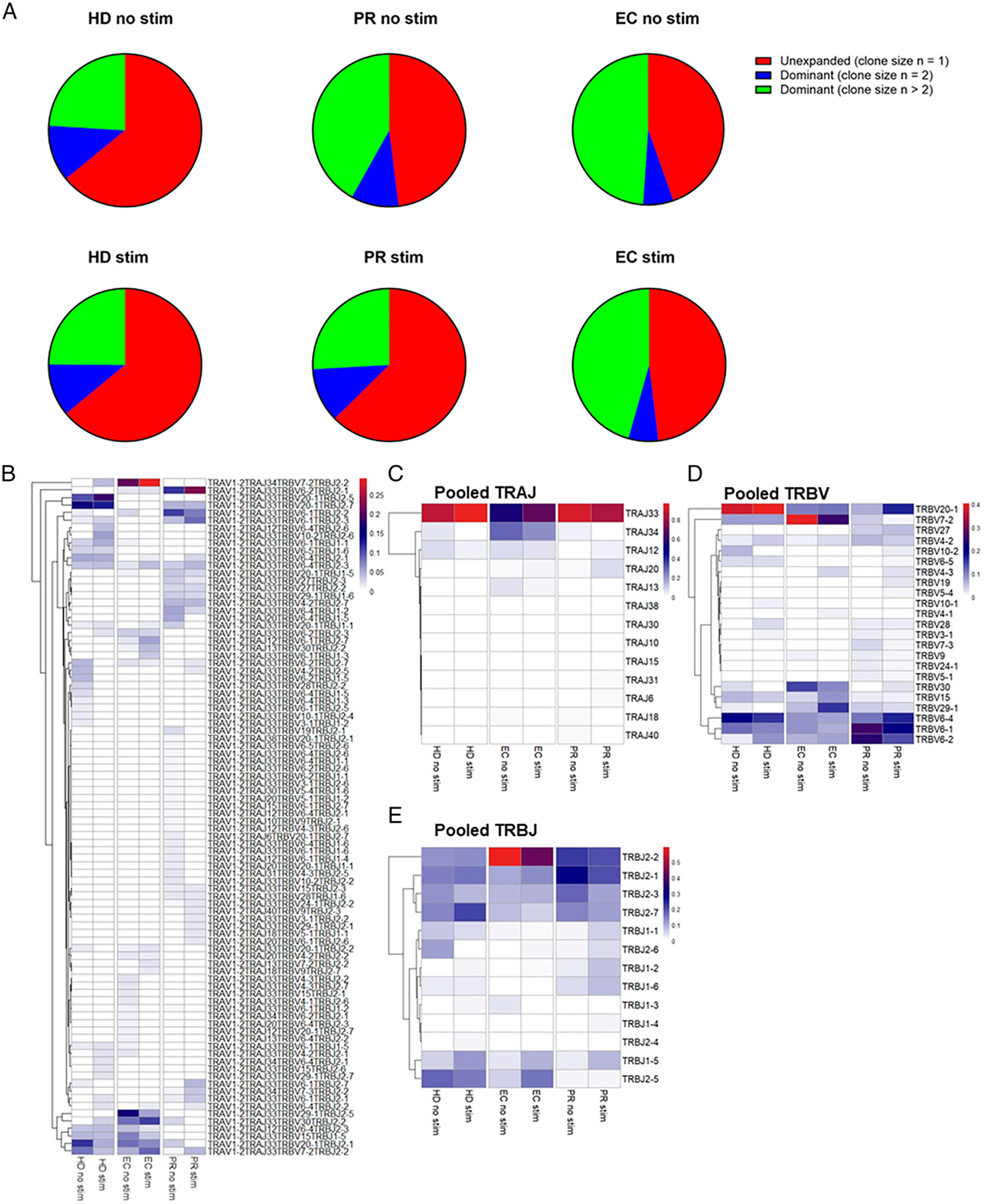
TCR usage in MAIT cells is different in HIV infection. MAIT cells were sorted from unstimulated and E. coli–stimulated PBMCs from four HD, four PR on ART, and three HIV-infected EC, and TCR was analyzed at the single-cell level using Illumina MiSeq sequencing. (**A**) Pie charts showing percentage frequency of unexpanded and expanded MAIT cell clones in each group. (**B**) TCR usage in unstimulated and in vitro–stimulated expanded MAIT cell clones obtained from pooling HD, PR, and EC individuals is shown as heatmap with hierarchical clustering performed using Euclidean distance. (**C**) TRAJ usage, (**D**) TRBV usage, and (**E**) TRBJ usage in all three groups is shown as heatmap.

Consistent with previous findings (35), we found that MAIT cell frequencies in peripheral blood were significantly lower in the PR and EC groups compared with the HD group (Supplemental Fig. 1). To examine the clonal distribution of MAIT cells in HIV, we stimulated PBMCs obtained from different donors (Table I) with the MR1 ligand-producing bacteria *E. coli*, sorted stimulated and unstimulated single TRAV1–2^+^ CD161^+^ MAIT cells, and obtained paired TCRα and TCRβ gene sequences. Any two or more MAIT cells expressing the same CDR3α and CDR3β regions (and thus TCRαβ pairing) were defined as dominant clones. We found that 35% of cells were part of dominant clones in unstimulated and stimulated cells in HD, 51% in unstimulated and 37% in stimulated MAIT cells of PRs, and 55% in unstimulated and 51% in stimulated cells of EC (Fig. 1A). We focused on dominant clones for our analysis because clones occurring at higher frequency suggests a proliferative response and also because more than one single cell is required for the reliable characterization of the gene expression profile of each clone (39,40). We observed unique clonal populations of MAIT cells with differences in TCRα and TCRβ usage among the three groups (Fig. 1B, Supplemental Fig. 2A). Consistent with previous findings (21, 40), we found the TRAJ and TRBV usage of MAIT cells was characterized by the preferential use of TRAJ33 and TRBV20–1, TRBV6–1, TRBV6–2, and TRBV6–4 in all HD (Fig. 1C, 1D). We note that two of three EC subjects preferentially used TRBV7–2 and TRBJ2–2 (Fig. 1D, 1E; Supplemental Fig. 2C), although our small sample size precluded statistical testing. When the top three most high-frequency (dominant) clones were compared in each condition across all groups, we found that in *E. coli*–stimulated conditions, select clones within HIV-infected subjects were expanded to a much larger extent than others, including TRAJ34 TRBV7–2 TRBJ2–2 in ECs (21% in unstimulated versus 29% with stimulated) and TRAJ33 TRBV6–2 TRBJ2–1 in PRs (11–22%) (Table II, Supplemental Fig. 2). Interestingly, EC and HD shared one dominant clone (TRAV1–2 TRAJ33 TRBV7–2 TRBJ2–2) in unstimulated condition and three dominant clones (TRAV1–2 TRAJ33 TRBV30 TRBJ2–2, TRAV1–2 TRAJ33 TRBV7–2 TRBJ2–2, and TRAV1–2 TRAJ34 TRBV7–2 TRBJ2–2) in stimulated conditions (Table III). In contrast, there were no other shared clones among PRs and the other groups in stimulated conditions. These data suggest that certain clones of MAIT cells in HIV patients may be more primed to expand upon bacterial stimulation, and further work is warranted to examine the functional significance of this observation.

### EC have different amino acid distribution in CDR3 region of MAIT cells compared with PRs and HDs

**FIGURE 2.**
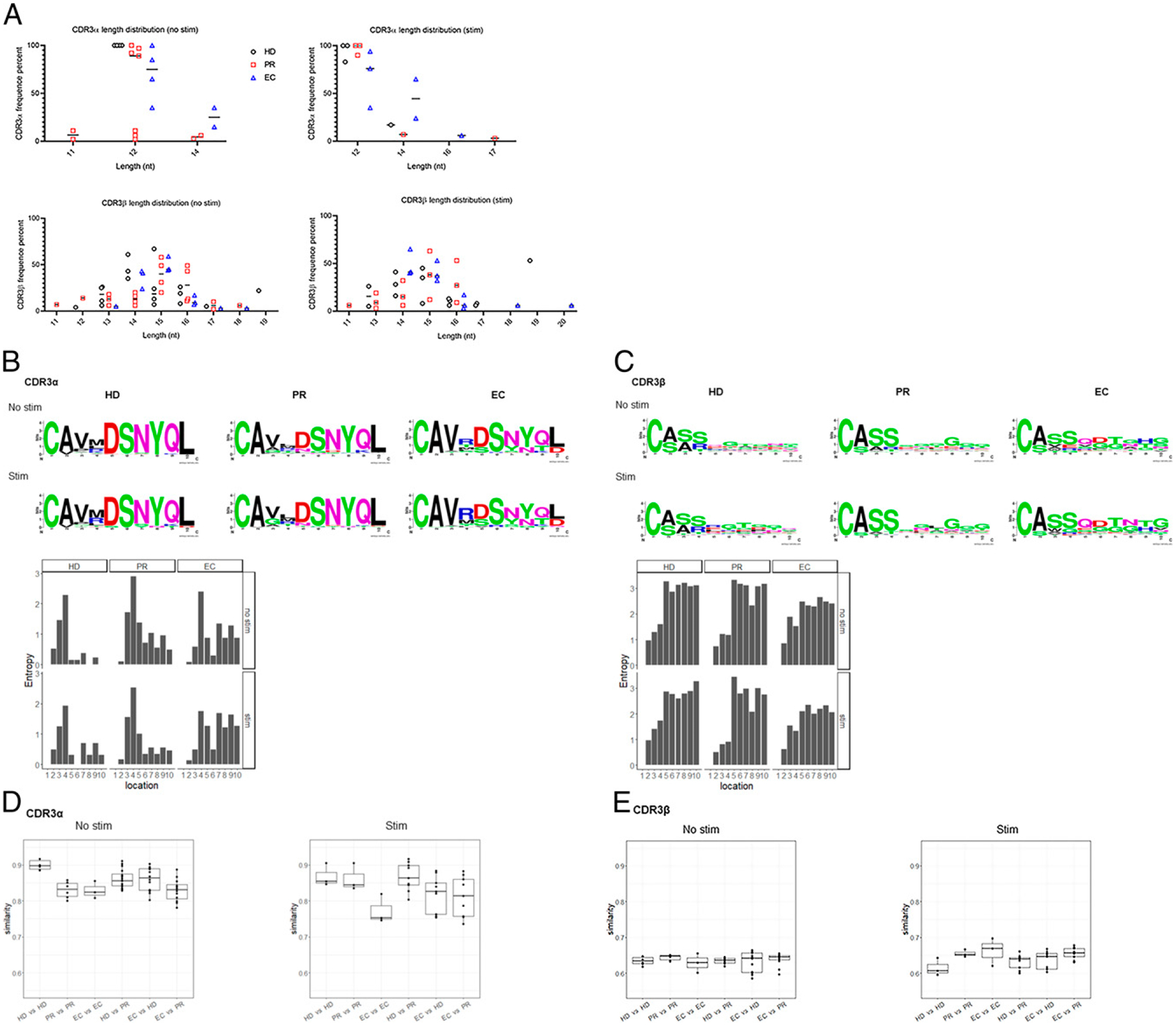
MAIT CDR3 region displays length and sequence diversity in HIV infection. The length distribution of CDR3α and CDR3β sequences from all donors in each group was analyzed. (**A**) Graphs depict CDR3α and CDR3β lengths observed in HD (black circle), PR (red squares), and EC (blue triangle) groups. Each symbol represents individual donor. (**B**) Visual representation of amino acid enrichments at each position across the CDR3α core compiled from expanded MAIT cell clones in each group. Entropy for distribution of amino acid at each of the 10 sites of HD, PR, and EC groups before and after stimulation was calculated, as described previously (38). (**C**) Visual representation of amino acid enrichments at each position and Shannon entropy analysis across the CDR3β core compiled from expanded MAIT cell clones in each group. CDR3α and CDR3β sequences from all donors in each group were pooled, and analysis was confined to sequences with a length of 10 aa. Graphics were generated using Seq2Logo. (**D** and **E**) CDR3α and CDR3β sequence similarity between each pair of subjects in a group (intragroup: HD versus HD, PR versus PR, and EC versus EC) and between each group (intragroup: HD versus EC, EC versus HIV, and HD versus HIV) before and after stimulation, as described previously (W.-J. Shen et al., manuscript posted on arXiv, 1205.6031).

To determine whether the differences observed in MAIT TCR usage were occurring at the clonotypic level, we investigated the length distribution and diversity of amino acids in the CDR3 regions. As observed in Fig. 2A, in HDs, CDR3αlength was 12 nt in both unstimulated and stimulated conditions; in PRs, it varied from 11–14 nt in unstimulated and 12, 14, and 17 nt in stimulated conditions; and in ECs, it was 12 or 14 nt in unstimulated condition or 12, 14, and 16 nt in stimulated conditions. CDR3β length varied from 11 to 19 nt in unstimulated and 11 to 20 nt in stimulated conditions for all three groups. Next, we generated amino acid distributions using the WebLogo application (http://weblogo.berkeley.edu/logo.cgi) and expressed the variability at the given positions of each CDR3α and CDR3β sequence as Shannon entropy (38). Interestingly, we found that in both unstimulated and stimulated conditions, MAIT cell CDR3α region in EC were mostly occupied by arginine (R) at fourth position, whereas PR had leucine (L) and HD had methionine (M) at the same position (Fig. 2B, Supplemental Fig. 3). Notably, all donors contain the Tyr95α residue (tyrosine [Y] at position 8) thought to be essential for MAIT cell activation (41–43) (Fig. 2B). The examination of Shannon entropies revealed that CDR3α positions 7–10 showed greater diversity in EC compared with HD and PR. In addition, amino acid residues at CDR3β positions 5–10 showed greater diversity in all three groups compared with amino acid residues at positions 1–4 (Fig. 2C, Supplemental Fig. 3). Collectively, we found distinct EC-specific CDR3 clonotype expansions within the MAIT cell population. We also compared full--length amino acid sequence similarity intragroup (between each pair of subjects of each group) and intergroup (between each pair of subjects of three different groups) as described previously (W.-J. Shen et al., manuscript posted on arXiv, 1205.6031). Fig. 2D shows overall similarity in CDR3α region is high, given that a value of 1 represents 100% similarity, and in both unstimulated and stimulated conditions, similarity within each donor in the HD group is greater than similarity within each donor in EC and PR groups. In addition, in unstimulated conditions, CDR3α region similarity between the EC and HD group trended to be higher than similarity between the EC and PR group (Fig. 2D). Fig. 2E shows similar patterns of CDR3β sequence similarity intragroup and intergroup in unstimulated condition, and upon stimulation, amino acid distributions within the HD group (intragroup) appears to be different from other intragroups and intergroups. Overall, we found different patterns of CDR3 amino acid distributions among the three groups, but our small sample size limited our ability to make further conclusions to our observations.

### MAIT cell phenotypic characteristics in HIV infection

**FIGURE 3.**
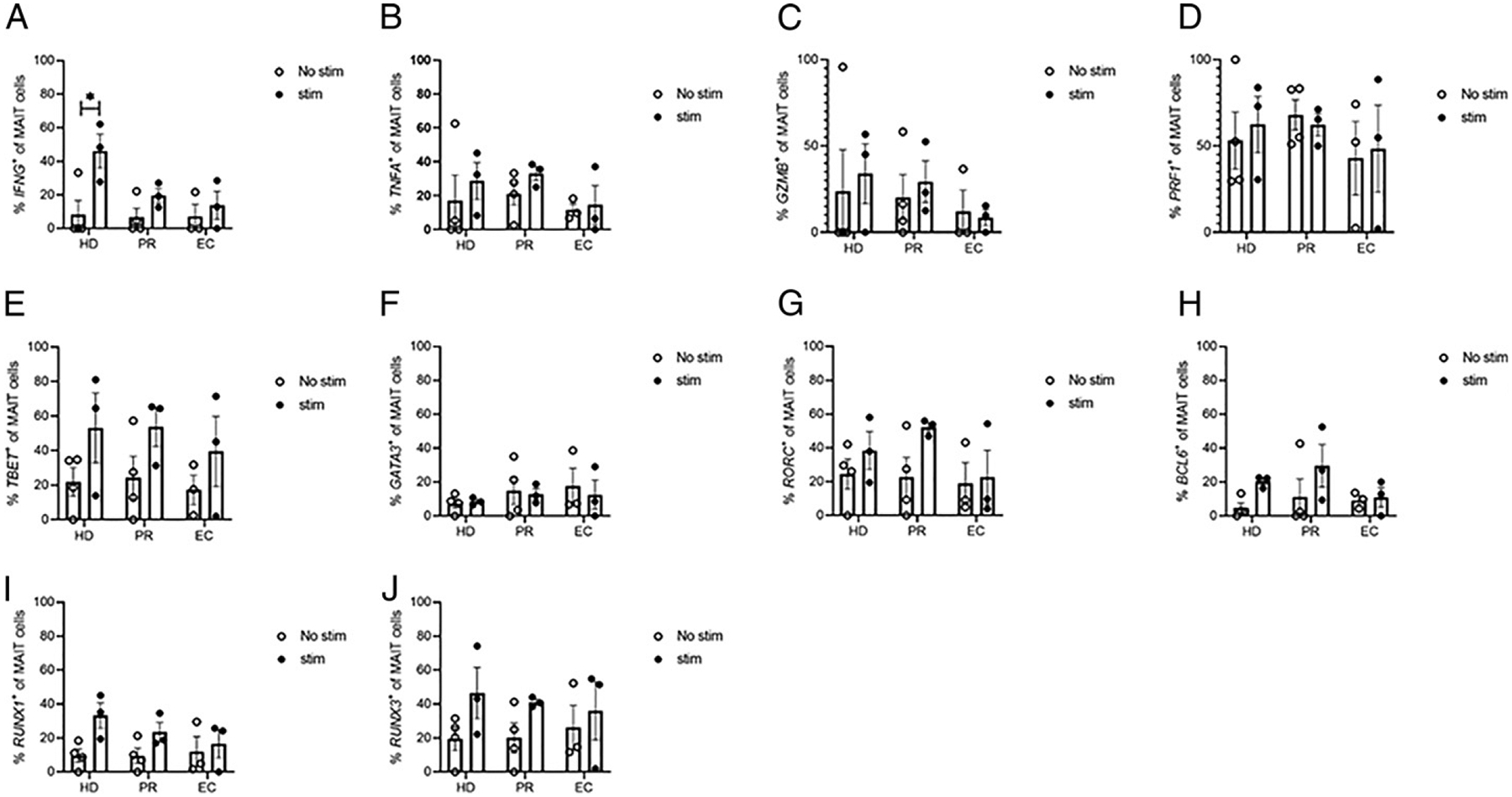
MAIT cell phenotype is different in HIV infection. MAIT cell TCRα and TCRβ genes and functional genes of interest were simultaneously sequenced. Bar graphs represent percentage of all the dominant MAIT cell clones expressing (**A**) *IFNG*, (**B**) *TNFA*, (**C**) *GZMB*, (**D**) *PRF1*, (**E**) *TBET*, (**F**) *GATA3*, (**G**) *RORC*, (**H**) *BCL6*, (**I**) *RUNX1*, and (**J**) *RUNX3* genes in HD, HIV, and EC. Data were analyzed using two-way ANOVA with Tukey multiple comparisons test.

To further characterize the MAIT cell function during HIV infection, in addition to TCR sequencing, we simultaneously measured multiple genes of interest (cytokines and transcription factors) from single MAIT cells. A previous study has shown that MAIT cell cytokine responses to stimulation with bacteria, such as *E. coli*, are impaired in chronic HIV-1 infection and partly recover during combination ART (34). Consistent with this, we observed that upon in vitro stimulation of PBMCs with fixed *E. coli*, higher frequencies of MAIT cells in HD express *IFNG* (*p* <0.05), whereas such differences were not statistically significant in EC or PR groups (Fig. 3). We did not see any statistically significant differences between groups in transcription of other known MAIT effector molecules, including *TNF, GZMB*, or *PRF1*, although our analysis was limited by small sample size. Similarly, expression of transcription factors (*TBET, GATA3, RORC, BCL6, RUNX1*, and *RUNX3*) also showed no significant differences in response to stimulation between the groups (Fig. 3).

## DISCUSSION

Disturbances in MAIT cell frequency and function have been described during HIV infection. However, the clonal distribution and associated phenotype are unknown. In this study, we evaluated the ex-vivo TCR repertoire and phenotype of MAIT cells at a single-cell level in HIV-1 infection. We found substantial diversity and heterogeneity in TCR usage by MAIT cells, demonstrated unique abilities of certain dominant MAIT clones from HIV-infected individuals to expand with *E. coli* stimulation, and found a more-unique distribution of clones among EC compared with PR and HD. These results suggest that MAIT TCR usage and proliferative patterns is different in HIV-1 infection and also raises the question of whether the unique clonality found in EC have functional relationships with the endogenous viral control observed in EC.

In this study, we expand on previous knowledge of MAIT cell clonality [mostly limited to descriptions of TRBV usage (44, 24)] by examining paired TCRα and TCRβ of MAIT cells from HIV patients at the single-cell level. Historically, initial identification of MAIT cells was associated with the expression of a canonical semi-invariant TRAV1–2/TRAJ33 TCR α-chain (20, 45). However, recent studies have shown that the TCRα repertoire of MAIT cells is more diverse than previously described (21, 28). Gold et al. (23) showed that pathogen-reactive MAIT cells frequently expressed additional TRAJ genes, namely TRAJ33, TRAJ12, and TRAJ20 genes. In our study, although the canonical TRAV1–2/TRAJ33 rearrangement was the predominant one featured in the TCR α-chain repertoire in HD and PRs, a clone of TRAV1–2/TRAJ34–expressing noncanonical MAIT cells dominated in one of the EC subjects, confirming that TRAV1–2 can use alternative TRAJ genes to generate MAIT TCR. Similar to TRAJ33, TRAJ34 gene also encodes Tyr95α residue in MAIT cells, thought to be essential for MR1-restricted ligand recognition (41–43). It has been shown that MAIT TCR α-chains are not exclusively germline encoded (45, 46) and most often contain single nucleotide additions that contribute to CDR3 core diversity (47); in this study, we found single amino acid change within the CDR3α sequences of PRs and ECs compared with HD. TCR α-chain is important for contact with MR1 (42), and how this single amino acid change in CDR3α impacts MAIT-TCR–MR1 contact and subsequent MAIT cell activation in HIV infection needs to be further studied.

The MAIT cell TCR repertoires are characterized in humans by varied TCR β-chain usage, with *TRBV20–1* and *TRBV6* family genes being predominantly used (20, 21, 45). Although the most expanded clones in PRs and HDs frequently displayed *TRBV20–1* and *TRBV6* family genes in our study, *TRBV7–2*, *TRBV29–1*, and *TRBV30* gene transcripts were also detected in the top three dominant clones of EC, suggesting greater MAIT TCR heterogeneity among EC. The presence of additional heterogeneity in MAIT cell TRBV usage has been reported before in different pathogen-specific responses (23) and in cancer (48), although this is the first study (to our knowledge) examining heterogeneity pairing TCRα with TCRβ usage. In HIV-1 infection, the effect of acute HIV-1 infection was associated with enhanced diversity of the CDR3 clonal distribution of both TCR α- and β-chains (27). We also detected diverse amino acid distribution in CDR3β loop in all groups; as CDR3β loop is positioned above the MR1 ligand-binding groove, it is suggested that the CDR3β loop can contribute to ligand discrimination (42, 49). Furthermore, the TCR β-chain expressed by MAIT cells has been shown to influence their MR1-dependent responses to microbial Ags (24, 50); therefore, it is possible that differences in microbial interactions in EC and PRs are associated with differential expansion of MAIT cell clones depending on the TCR β-chain used. It can be hypothesized that the unique expanded MAIT clones observed in ECs (such as TRAV1–2 TRAJ34 TRBV7–2 TRBJ2–2) have greater functional avidity of their TCR to MAIT cell ligands and contribute to cytotoxic response and endogenous viral control observed in EC. Further studies are needed to compare and contrast the functional advantage of the most expanded MAIT clonotypes in ECs and PRs.

MAIT cells are functionally impaired in chronic HIV patients (34), findings that are in line with our MAIT cell phenotypic data in which we found a trend of lower frequencies of *IFNG*-expressing MAIT cells in EC and PRs compared with HD. We did not observe differences between MAIT cell expression of cytotoxic molecules and transcription factors of PRs and ECs.

Our study has several limitations. First, because of our use of the TRAV1–2 Ab (the MR1 tetramer was not available during our study period) for sorting, our analysis was restricted to TRAV1–2 MAITs. Second, the methodology we used can couple TCR sequence with transcriptional profiling of single cells, but we cannot quantify individual gene expression. Third, we could not evaluate protein expression, and acquisition of protein expression data along with transcriptome data using cellular indexing of transcriptomes and epitopes by sequencing (CITE-seq) (51) or by using chromium next gel bead-in emulsions technology with barcoding for cell surface protein (10X genomics) would be useful in future studies. Fourth, although we found interesting functional differences in ECs and non-ECs compared with HDs, the study was an exploratory one that was not powered to detect differences. Furthermore, we were not able to recruit HIV^+^ individuals who are rapid progressors, with viral loads >30, and evaluate MAIT TCR clonality and phenotype in them. Fifth, we found differences in TCR repertoire between groups upon in vitro *E.coli* stimulation, but we have not addressed how TCR-independent cytokine stimulation influences MAIT TCR repertoire in HIV infection. Last, we do not understand functional implications of preferential expansion of certain MAIT clonotypes in EC, and future experiments should assess whether MAIT cell clonotypes that expand in EC may play a protective role in HIV infection or subsequent secondary infections. Also, in our study, MAITs were obtained from peripheral blood, and whether MAIT cell clonality differs at mucosal sites is not known.

Nonetheless, to our knowledge, this is the first report of MAIT single-cell paired clonality and functionality sequencing data in HIV-1 infection. We show unique clonal populations of MAIT cells and different amino acid distribution in CDR3 region among EC compared with PR and HD. Our findings suggest that MAIT cells harbor multiple layers of heterogeneity in response to HIV-1 infection and may contribute to recognition and clearance of HIV-1–infected cells.

## Supplementary Material

Supplementary data

## Figures and Tables

**TABLE I.** MAITs were sorted from the following subjects for TCR and phenotyping analysis at a single-cell level using Illumina Miseq platform

Subject ID	Age	Sex	Donor Category	CD4^+^ T Cell Countin Cells/μl (%)	Viral Load (Copies/ml)
EC 1	43	Male	EC	564 (27)	<30
EC 2	62	Male	EC	618 (24)	<30
EC 3	68	Male	EC	601 (27)	<30
PR 1	62	Female	Chronic HIV on ART	169 (16)	<30
PR 2	51	Male	Chronic HIV on ART	523 (28)	<30
PR 3	56	Male	Chronic HIV on ART	881 (34)	<30
PR 4	61	Male	Chronic HIV on ART	524 (31)	<30
HD 1	38	Male	Healthy donor	—	—
HD 2	40	Male	Healthy donor	—	—
HD 3	39	Male	Uninfected, on PrEP	—	—
HD 4	53	Male	Healthy donor	—	—

ID, identifier; PrEP, pre-exposure prophylaxis (Truvada).

**TABLE II.** List of high-frequency (dominant) clones in HD, PR, and ECs

				Unstimulated			Stimulated		
Subject	TCR Usage	CDR3α	CDR3β	*n* (Number of Expanded Clones)	Total Expanded Clones	% Total Expanded Clones	*n* (Number of Expanded Clones)	Total Expanded Clones	% Total Expanded Clones
HD	TRAV1-2TRAJ33TRBV20-1TRBJ2-5	CVPMDSNYQLIW	CSARLGTPNQAGVQETQYF	12	139	9	11	104	11
	TRAV1-2TRAJ33TRBV20-1TRBJ2-7	CAVLDSNYQLIW	CSATRGPDFYEQYF	12	139	9	8	104	8
	TRAV1-2TRAJ33TRBV20-1TRBJ2-1	CAVLDSNYQLIW	CSARDVAGDSYNEQFF	10	139	7	2	104	2
	TRAV1-2TRAJ33TRBV10-2TRBJ2-6	CAVRDSNYQLIW	CASSETEDGANVLTF	0	139	0	6	104	6
	TRAV1-2TRAJ33TRBV20-1TRBJ2-5	CAVRDSNYQLIW	CSARLGTPNQAGVQETQYF	12	139	9	11	104	11
PR	TRAV1-2TRAJ33TRBV6-2TRBJ2-1	CAGLDSNYQLIW	CASSSNLGGGGDEQFF	25	237	11	26	117	22
	TRAV1-2TRAJ33TRBV6-1TRBJ2-2	CAVTNSNYQLIW	CASSDGTGHTGELFF	26	237	11	9	117	8
	TRAV1-2TRAJ20TRBV6-4TRBJ1-5	CAVVLGDYKLSF	CASSSTGEGNQPQHF	12	237	5	0	117	0
	TRAV1-2TRAJ33TRBV6-1TRBJ2-7	CAGLDSNYQLIW	CASSIGLGSSYEQYV	0	237	0	5	117	4
	TRAV1-2TRAJ33TRBV6-1TRBJ2-3	CAVTDSNYQLIW	CASSPLAGADTQYF	4	237	2	5	117	4
	TRAV1-2TRAJ33TRBV4-2TRBJ2-7	CAVRDSNYQLIW	CASSQEGASSYEQYF	8	237	3	5	117	4
EC	TRAV1-2TRAJ34TRBV7-2TRBJ2-2	CAVRSSYNTDKLIF	CASSQDTNTGELFF	43	205	21	43	148	29
	TRAV1-2TRAJ33TRBV29-1TRBJ2-5	CAVTDSNYQLIW	CSVEVGTAHSETQYF	33	205	16	8	148	5
	TRAV1-2TRAJ33TRBV20-1TRBJ2-1	CAVSDSNYQLIW	CSARSPGTHNEQFF	17	205	8	10	148	7
	TRAV1-2TRAJ33TRBV30TRBJ2-2	CAVRDSNYQLIW	CAWGQGGGHVGELFF	4	205	2	12	148	8
	TRAV1-2TRAJ33TRBV7-2TRBJ2-2	CAVRDSNYQLIW	CASSQDTNTGELFF	10	205	5	11	148	7

**TABLE III.** MAIT cell expanded closes shared between HD, PR, and ECs

Subjects	Shared Clones
No stimulation	
HD and PR	None
PR and EC	TRAV1-2 TRAJ33 TRBV30 TRBJ2-2
	TRAV1-2 TRAJ33 TRBV6-2 TRBJ2-1
EC and HD	TRAV1-2 TRAJ33 TRBV7-2 TRBJ2-2
All three	None
Stimulation	
HD and PR	None
PR and EC	None
EC and HD	TRAV1-2 TRAJ33 TRBV30 TRBJ2-2
	TRAV1-2 TRAJ33 TRBV7-2 TRBJ2-2
	TRAV1-2 TRAJ34 TRBV7-2 TRBJ2-2
All three	None

## Abbreviations used in this article:

ART: antiretroviral therapy
EC: elite controller
HD: non-HIV–infected donor
MAIT: mucosal-associated invariant T
NEB: New England Biolabs
PR: HIV-infected progressor
