# Supplementary material for: Diverse Mucosal-Associated Invariant TCR Usage in HIV Infection
Source: Immunohorizons. Author manuscript; Available in PMC 2023 Oct 10. (PMC10563122; doi:10.4049/immunohorizons.2100026)
Supplement: Supplementary data [file NIHMS1933253-supplement-Supplementary_data.pdf]

Supplementary Figure 1

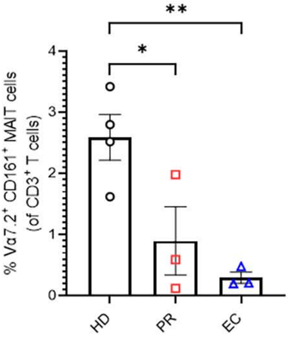

Supplementary Figure 1: Circulating MAIT cell frequencies in HD, PR and ECs. PBMCs were isolated from individual donors and analyzed using flow cytometry. MAIT cells were gated on live CD3+ Vα7.2+ CD161+ cells. Each symbol represents an individual subject. Statistical significance was assessed using one-way ANOVA and Tukey's multiple comparison test. * denotes $p \leq 0.05$ and ** denotes $p \leq 0.01$

aai.org/immunohorizons/article-supplement/234006/pdf/ih_2100026_supplemental_1/ by University of

Supplementary Figure 2

(A) Individual TCR

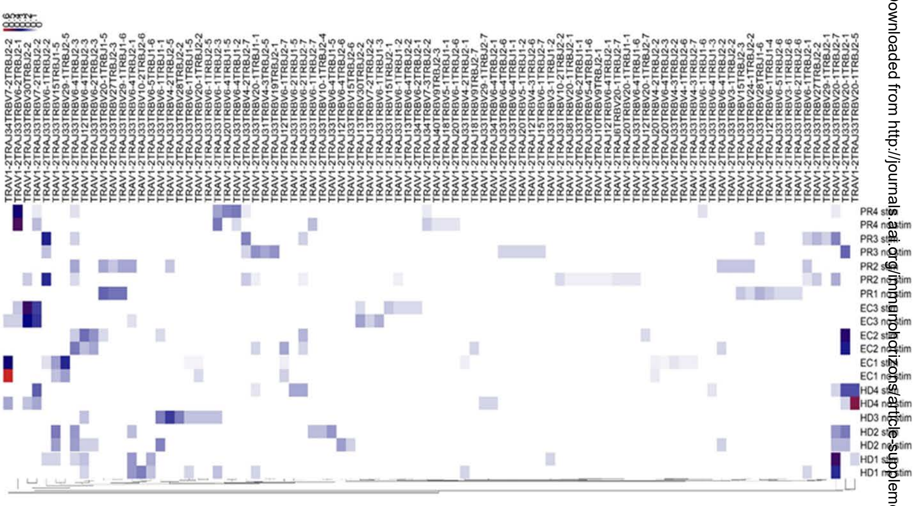

(B) Individual TRAJ

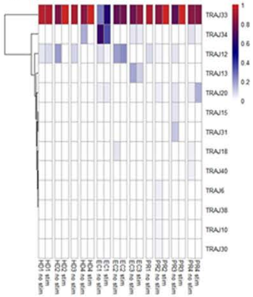

(C) Individual TRBV

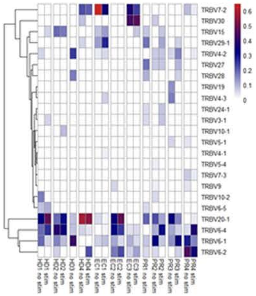

(D) Individual TRBJ

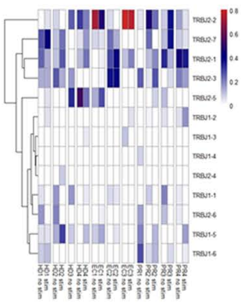

Supplementary Figure 2: TCR including TRAJ, TRBV and TRBJ usage in expanded MAIT clones of individual donors. The weighted (A) TCR, (B) TRAJ, (C) TRBV and (D) TRBJ usage profile for HD, PR and EC individual donors are shown as a heatmap with hierarchical clustering performed using Euclidean distance.

Downloaded from http://journals.aai.org/immunohorizons/article-supplement/234006/pdf/ih_2100026_supplemental_1/ by University of Utah user on 22 September 2023

Supplementary Figure 3

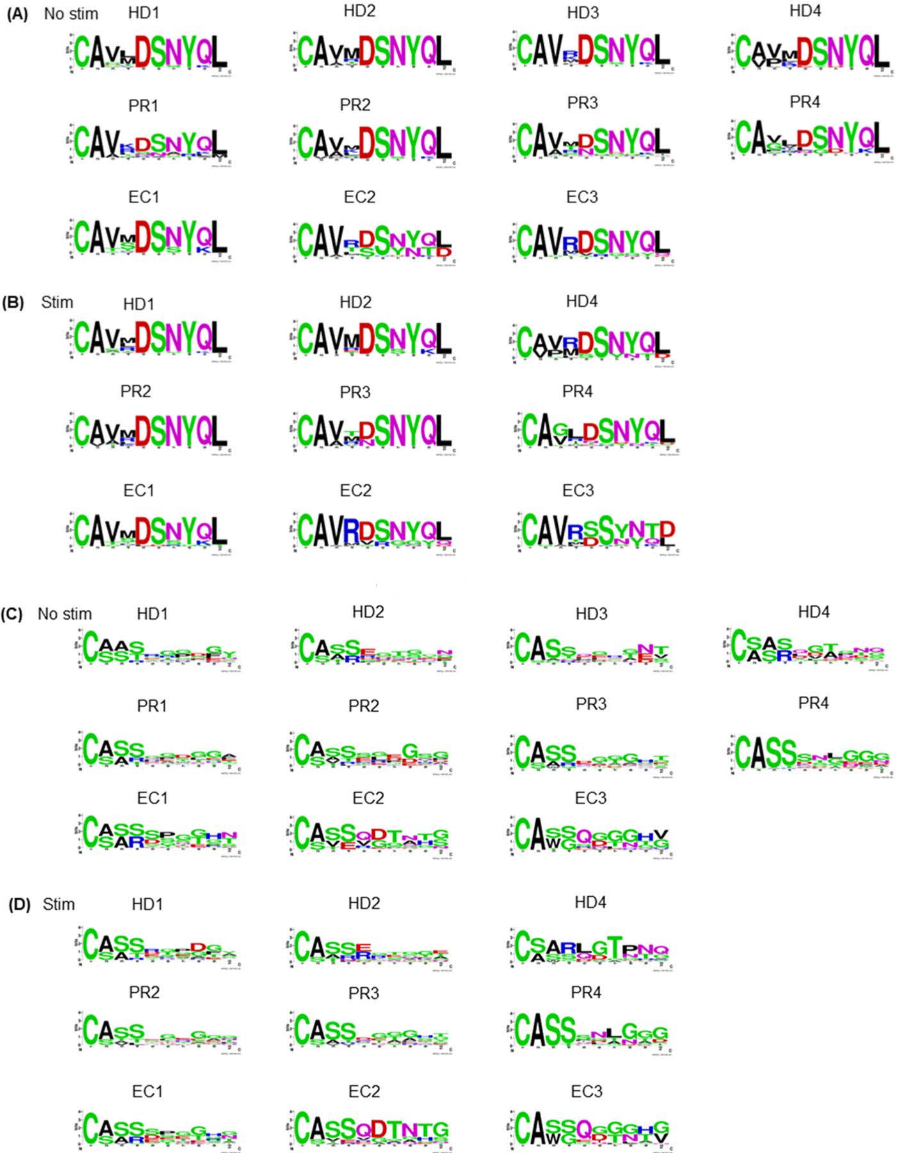

Supplementary Figure 3: MAIT CDR3α and CDR3β sequences in HD, PR and EC individual donors. Visual representation of amino acid enrichments at each position across the CDR3α (A and B) and CDR3β (C and D) compiled from unstimulated and stimulated expanded MAIT cell clones in each donor. Analysis was confined to sequences with a length of 10 amino acids. Graphics were generated using Seq2Logo.

Downloaded from http://journals.aai.org/immunohorizons/article-supplement/234006/pdf/ih_2100026_supplemental_1/ by University of Utah user on 22 September 2023
